# From Prokaryotes to Eukaryotes: Insights Into the Molecular Structure of Glycogen Particles

**DOI:** 10.3389/fmolb.2021.673315

**Published:** 2021-04-29

**Authors:** Qing-Hua Liu, Jia-Wei Tang, Peng-Bo Wen, Meng-Meng Wang, Xiao Zhang, Liang Wang

**Affiliations:** ^1^State Key Laboratory of Quality Research in Chinese Medicines, Macau University of Science and Technology, Macau, China; ^2^Faculty of Chinese Medicine, Macau University of Science and Technology, Macau, China; ^3^Department of Bioinformatics, School of Medical Informatics and Engineering, Xuzhou Medical University, Xuzhou, China; ^4^Jiangsu Key Laboratory of New Drug Research and Clinical Pharmacy, School of Pharmacy, Xuzhou Medical University, Xuzhou, China

**Keywords:** glycogen structure, chain length, fragility, α particle, diabetes mellitus

## Abstract

Glycogen is a highly-branched polysaccharide that is widely distributed across the three life domains. It has versatile functions in physiological activities such as energy reserve, osmotic regulation, blood glucose homeostasis, and pH maintenance. Recent research also confirms that glycogen plays important roles in longevity and cognition. Intrinsically, glycogen function is determined by its structure that has been intensively studied for many years. The recent association of glycogen α-particle fragility with diabetic conditions further strengthens the importance of glycogen structure in its function. By using improved glycogen extraction procedures and a series of advanced analytical techniques, the fine molecular structure of glycogen particles in human beings and several model organisms such as *Escherichia coli*, *Caenorhabditis elegans*, *Mus musculus*, and *Rat rattus* have been characterized. However, there are still many unknowns about the assembly mechanisms of glycogen particles, the dynamic changes of glycogen structures, and the composition of glycogen associated proteins (glycogen proteome). In this review, we explored the recent progresses in glycogen studies with a focus on the structure of glycogen particles, which may not only provide insights into glycogen functions, but also facilitate the discovery of novel drug targets for the treatment of diabetes mellitus.

## Introduction

**FIGURE 1:**
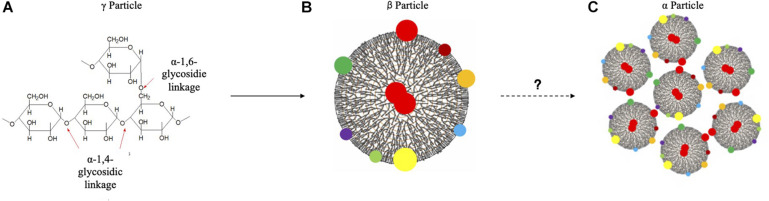
Schematic illustration of eukaryotic glycogen particles. **(A)** Short chain oligomers. **(B)** β particle. **(C)** α particle. Glycogen sub-proteomes were denoted by colored dots. Red dots were glycogenin, which were distributed in the center of and on the surface of β particles. Question mark indicates the unknown assembly mechanism of glycogen α particles.

Glycogen is a highly branched polysaccharide that is widely distributed across species from prokaryotes to eukaryotes (Wilson et al., 2010), which plays pivotal roles in a variety of extremely important functions, such as energy reserve (Greenberg et al., 2006), osmotic pressure maintenance (Brown, 2004), host colonization (Jones et al., 2008), blood glucose homeostasis (Han et al., 2016), pH maintenance (Fredricks et al., 2014) and tumor development (Zhang et al., 2020). Since its first discovery by the French physiologist Claude Bernard in 1857, the structure and function of glycogen have been extensively characterized over the centuries, which leads to the grants of multiple Nobel prizes (Brewer and Gentry, 2019). Although glycogen has multi-facet roles, its function is intrinsically determined by its unique structure (Meléndez-Hevia et al., 1993; Wang and Wise, 2011). In general, the structure of glycogen particles could be divided into three levels: level (1) short-chain oligomers; level (2) spherical β particles with an average diameter of 20 nm; and level (3) large rosette-shaped α particles aggregated together by β particles, the diameter of which ranges roughly up to 300 nm (Li and Hu, 2019). It has also been proposed that level 1 structure is a roughly 3 nm protein-rich γ particle that is highly electro-condense (Prats et al., 2018). However, glycogen γ particles are typically used in the old literature and does not represent particles *per se*. The general procedures of glycogen particle formation in eukaryotes include the following steps. Firstly, glycogen biogenesis is initiated by glycogenin (GYG), which is a self-glycosylation enzyme (Whelan, 1986). It is worth noting that, in human beings and most mammals, there are two isoforms of glycogenin, widely-expressed isoform glycogenin 1 (GYG1) and liver-predominant isoform glycogenin 2 (GYG2); however, only a single GYG gene is expressed in all tissues in rodents (Zhai et al., 2001; Testoni et al., 2017). After initiation, glycogen synthase is then recruited to elongate oligosaccharide chains via α-1, 4-glycosidic bonds in the linear chains, which is further branched by glycogen branching enzyme via α-1, 6-glycosidic bonds (Wang and Wise, 2011). Repetition of the procedure leads to the formation of β particles (Greenberg et al., 2006). As for the higher structural level, β particles are aggregated together to form the rosette-shaped α particles via currently unknown mechanisms. In terms of the formation of glycogen particles in prokaryotes, similar steps are involved except that no glycogenin is needed during the initiation stage (Ugalde et al., 2003). In addition, a small amount of proteins are associated with eukaryotic β glycogen particles to form sub-cellular proteomes known as glycogen proteome (Stapleton et al., 2010). Glycogen-associated proteins were also reported in bacteria (Cardona et al., 2001). However, whether glycogen particles from prokaryotes have similar sub-proteomes are sparsely studied, which is worthy of further investigation. A schematic illustration is present below to summarize the three structural hierarchies of glycogen particles in eukaryotes (Figure 1).

**FIGURE 2:**
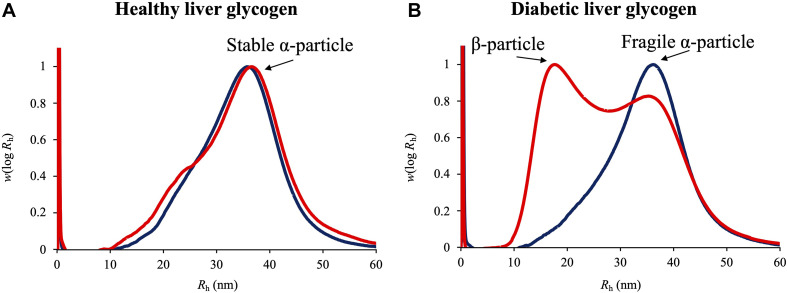
Weight distributions as functions of molecular size of glycogen particles in healthy and diabetic liver glycogen extracted from *Rat rattus*. **(A)** Healthy liver glycogen. **(B)** Diabetic liver glycogen. Glycogen α and β particles were annotated and designated with black arrows. Dark blue curve: liver glycogen sample in original state. Red curve: liver glycogen sample treated with DMSO.

Glycogen α particle was initially discovered in rat liver by Pierre Drochmans in 1962 via transmission electron microscopy (TEM) images (Drochmans, 1962). After that, its functions and assembly mechanisms have been widely studied. Recently, the structure of liver glycogen α particles has been reported to be associated with the development of type 2 diabetes (Sullivan et al., 2012; Besford et al., 2015; Deng et al., 2015). A couple of elegant studies showed that the structure of glycogen α particles in healthy liver followed a diurnal alteration between fragility and stability, while the rhythm was disrupted in diabetic liver, where glycogen α particles were constantly fragile (Figure 2; Hu et al., 2018, 2019). *In vitro* biochemical analysis and theoretical studies revealed that fragile α particles could be quickly disintegrated into β particles in the presence of dimethyl sulfoxide (DMSO), which were then degraded into glucose in a faster pace (Powell et al., 2015), indicating that molecularly fragile liver glycogen could exacerbate poor blood glucose control in diabetes. Previously, a study on tapeworm glycogen revealed that β particles had high rate of glycogen turnover than α particles, which indicated that glycogen α particle was more likely to be an energy reserve while β particle was a source of readily mobilizable carbohydrate (Lumsden, 1965). A couple of recent experimental and theoretical studies also validates that α particles degrade more slowly than β particles (Besford et al., 2015; Jiang et al., 2016).

How β particles aggregate to form α particles has not been elucidated yet due to the complexity of glycogen metabolism in eukaryotes, especially in animals and human beings. Recently, Liu et al. (2020a) characterized the fine structure of glycogen particles in *Caenorhabditis elegans*, a lower form of life and routine laboratory model animal; this is also the first time that glycogen structural fragility was reported in worms. Since *C. elegans* is often used to study diabetes, it would be convenient to use the model animal to elucidate the assembly mechanisms of glycogen structure (Hanover et al., 2005; Zhu et al., 2016). For a long time, it was thought that prokaryotes only accumulated glycogen in the form of small β particles (Wang et al., 2019b). Morphological studies of glycogen particles from *Selenomonas ruminantium* and *Fibrobacter succinogenes* via transmission electron microscopy (TEM) showed the possible existence of rosette-shaped α particles in bacteria, though no much attention was given to the discovery (Kamio et al., 1981; Gong and Forsberg, 1993). Glycogen α particles were recently reported in *Mycobacterium tuberculosis*, *Streptomyces venezuelae*, and *Escherichia coli* via TEM and NMR analyses (Rashid et al., 2016). Through a newly developed and optimally mild extraction method, together with advanced analytical techniques, Wang et al. (2019b) isolated glycogen from *Escherichia coli*, a prokaryotic model organism, and confirmed, for the first time, the existence of both stable and fragile glycogen α particles in prokaryotes. The study could also provide us with insights into the assembly mechanisms of glycogen α particles.

Taken together, the presence of glycogen α particles across species from prokaryotes to eukaryotes indicates that the formation of glycogen α particles might be evolutionarily relevant. In addition, except for animal models such as mouse and rat, it would be possible for researchers to use other simpler organisms like *E. coli* and *C. elegans* to investigate the molecular mechanisms of α-particle assembly and fragility. In this review, we aim to provide a comparative and comprehensive overview of glycogen structure from β articles to α particles in different species. Insights from this review would facilitate our understanding of glycogen structural fragility, with potentials in developing novel drug targets for the treatment of diabetes.

## Glycogen β Articles

### Formation of Glycogen β Articles

**FIGURE 3:**
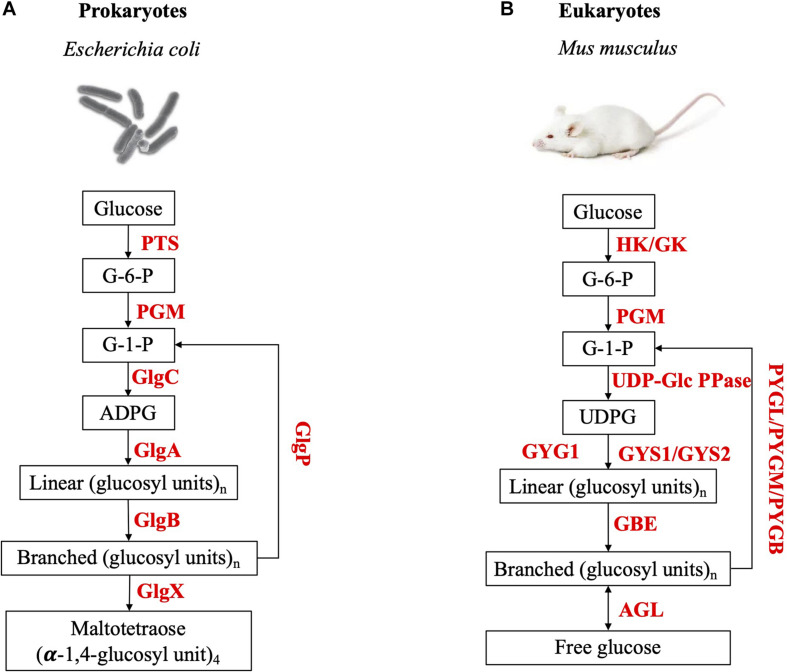
Illustration of the metabolism pathway of glycogen particles in **(A)** prokaryotes and **(B)** eukaryotes. PTS, Phosphoenol-pyruvate:sugar phosphotransferase system; PGM, Phosphoglucomutase; GlgC, Glucose-1-phosphate adenylyltransferase; GlgA, Glycogen synthase; GlgB, Glycogen branching enzyme; GlgP, Glycogen phosphorylase; GlgX, Glycogen debranching enzyme; HK, Hexokinase; GK, Glucokinase; UPD-Glc PPase, UTP-glucose-1-phosphate uridylyl-transferase; GYG, Glycogenin; GYS, Glycogen synthase; GBE, Glycogen branching enzyme; AGL, Glycogen debranching enzyme; PYGL, Glycogen phosphorylase (liver form); PYGM, Glycogen phosphorylase (muscle form); PYGB, Glycogen phosphorylase (brain form).

It is widely accepted that the *de novo* synthesis of glycogen β particles relies on the auto-glycosylation of a protein in both eukaryotes (Rodriguez and Whelan, 1985) and prokaryotes (Ugalde et al., 2003). In eukaryotes, glycogenin (GYG), a homo-dimer glycosyltransferase, initiates glucose polymerization at conserved tyrosine site (Tyr-195 in human GYG1, Tyr-230 in yeast GYG1, and Tyr-194 in *C. elegans* GYG) and extends the oligosaccharide chain up to seven glucosyl residues (Smythe and Cohen, 1991; Zeqiraj et al., 2014). Glycogenin and glycogen synthase (GS) are then complexed together to continue elongating the chains. After that, GYG and GS dissociates, while GS cooperates with glycogen branching enzyme to synthesize β particles (Zeqiraj et al., 2014). The mature glycogen β particles possesses covalently-bound glycogenin in its center (Smythe and Cohen, 1991; Prats et al., 2018). Different from eukaryotes, no glycogenin or its homologs has ever been detected in prokaryotes (Ugalde et al., 2003). Evidence from the study of *Agrobacterium tumefaciens* shows that glycogen synthase plays a primer role in the initiation during bacterial glycogen synthesis via self-glycosylation (Ugalde et al., 2003). However, glycogen synthase is not attached to the mature glycogen particle in its center (Ugalde et al., 2003). Recently, clinical studies challenged the essentiality of glycogenin in the formation of eukaryotic glycogen, according to which, patients with glycogenin-1 deficiency in muscle could still accumulate glycogen particles, with no up-regulation of the glycogenin-2 gene (Visuttijai et al., 2020). Experimental studies also revealed that glycogenin-deficient mice, which only has a single copy of the glycogenin gene in its genome, still accumulate high amounts of glycogen in striated muscle but the size is dysregulated (Testoni et al., 2017). Thus, this might suggest that glycogenin may be associating near or on the surface of the particles, rather than immediately at the center. Although glycogenin is dispensable in eukaryotes for the synthesis of the polysaccharide *in vivo*, glycogenin deficiency is a pathological state and causes polyglucosan storage (Visuttijai et al., 2020). In mice, glycogenin deficiency lead to lower resting energy expenditure and less resistance to endurance exercise (Testoni et al., 2017). A comparative illustration of glycogen metabolism pathways in prokaryotes and eukaryotes were present in Figure 3.

### Structural Models of Glycogen β Particles

Initially, Haworth et al. (1937) proposed that glycogen structure was in laminated form while Staudinger and Husemann (1937) suggested an alternative comb-like model. Later, Meyer et al. (1970) suggested an irregular, tree-like model to describe glycogen structure, which was further confirmed experimentally by Larner et al. (1952) in the Cori’s group. With the development of enzymology in glycogen metabolism, Gunja-Smith et al. (1970) revised the regularly re-branched glycogen model (Meyer’s Model) and proposed a tiered model with two types of chains, external A chains with no branches and internal B chains containing two branch-points, which was also known as the Whelan model. Based on this model, a series of experimental and mathematical studies revealed that the optimal structure of β particles had an average chain length of 13 degree of polymerization (DP), 2 branching points on each B chain, and 12 concentric tiers; thus, in a typical optimized β particle, the diameter is around 42 nm and a total of 55000 glucosyl residues exist within (Meléndez-Hevia et al., 1993). In addition, based on the Whelan model, constant branching leads to self-limiting of the size of glycogen β particles, and the particle density increases with the growth of tiers. By means of mathematical models, Meléndez et al. (1999) also investigated the possibility of whether glycogen possesses a fractal structure.

However, all these models have recently been proven incorrect by both theoretical simulation and experimental study, though the model by Melendez and co-workers was visually helpful for initially understanding glycogen structure in the 1990s. In specificity, the size of glycogen particles (radius of gyration, R_g_) has already been shown to scale as the log of the molecular weight log(Mw), that is, R_g_ ∼ log(Mw) by Rolland-Sabaté et al. (2008), which invalidates the fractal model assumption. Besford et al. (2015) used small angle X-ray scattering (SAXS) to analyze mouse β-particles, revealing these particles to be randomly branched polymers rather than hierarchical or fractal polymers. In addition, Zhang et al. (2018) also used Monte Carlo model to simulate glycogen β particle formation, which also generated randomly branched polymers.

### Chain-Length Distribution

As an important structural parameter, chain-length distribution correlates with other structural features of β particles such as percentage of branching points, particle sizes, and particle densities (Wang et al., 2020b). All together, these features have great impacts on glycogen metabolism and physiological functions. As an important energy compound in both prokaryotes and eukaryotes, glycogen is normally considered as a fast, transient, and flexible store of nutrients due to its highly branched structure (Meléndez et al., 1999). For example, liver glycogen decreases rapidly during fasting to maintain blood glucose homeostasis and keep energy supply (Han et al., 2016) while skeletal muscle glycogen can be rapidly depleted during exercise with a high rate of glycolysis. In addition, brain glycogen is also found to decreases rapidly during prolonged exercise via microwave irradiation measurement (Matsui et al., 2011). However, some studies indicated that chain length distribution could alter glycogen degradation rate (Wang and Wise, 2011; Wang et al., 2020b). In specificity, according to the hypothesis called durable energy storage mechanism, glycogen with a chain-length distribution biased toward shorter regions is more likely to be degraded in a slower rate, vice versa (Wang and Wise, 2011). The molecular mechanism behind the hypothesis is that hydrolysis of α-1,4- and α-1,6-glycosidic linkages are + 1,300 cal/mol (exothermic reaction) and -1,100 cal/mol (endothermic reaction) at 25°C, respectively. Thus, α-1,4-glycosidic linkages are much easier to be broken down than α-1,6-glycosidic linkages. In addition, glycogen branching process involves break of α-1,4-glycosidic linkages and generation of α-1,6-glycosidic linkages, which makes branching a thermodynamically-favored procedure during glycogen synthesis (Wang and Wise, 2011). Thus, glycogen with more short chains will have more branches, which requires higher energy to break down, leading to slower degradation rate.

**FIGURE 4:**
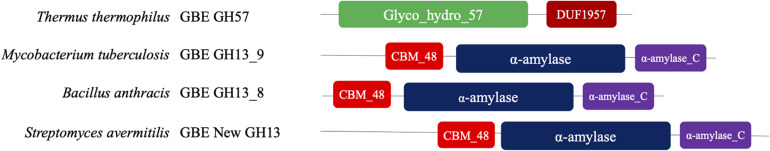
Schematic illustration of different types of glycogen branching enzymes. A typical GH57 GBE consists of two domains, Glyco_hydro_57 and DUF1957. A typical GH13 GBE consists of three domains, CBM48, α-amylase, and α-amylase_C, which is divided into three sub-groups based on the lengths of its N-terminus. Domain compositions were sourced from UniProt database. *Thermus thermophilus* (UniProt ID: Q5SH28). *Mycobacterium tuberculosis* (UniProt ID: P9WN45). *Bacillus anthracis* (UniProt ID: Q81K82). *Streptomyces avermitilis* (UniProt ID: Q82JF0).

The molecular mechanisms of chain length distributions in glycogen particles have been intensively studied. Many enzymes were reported to have impacts on this primary structural feature. One of the key enzymes that determine chain length distribution is glycogen branching enzyme (GBE), which normally cleaves 8-14 glucosyl residues of a glucan chain in an α-1,4-glucosidic linkage and transfers it within the same chain or to a neighboring chain via an α-1,6-glucosidic linkage (Froese et al., 2015). However, GBEs from different species might have different preferences for the lengths of transferred chains. Phylogenetically speaking, GBEs can be divided into two types, glycoside hydrolase family 13 (GH13) and glycoside hydrolase family 57 (GH57), which have distinct differences in terms of structure and mechanism of action (Palomo et al., 2011). It is noteworthy that the terminology GH13 and GH57 refers to The Carbohydrate Active EnZyme (CAZy) classification (Henrissat, 1991). In bacteria, GH13 GBE is the most common type while GH57 GBE is rarely seen and mainly identified in *Terrabacteria* and PVC (*Planctomycetes*, *Verrucomicrobia*, *Chlamydiae*) superphylum (Wang et al., 2019d). Two sub-groups of GH13 GBE were reported to exist in bacteria, GH13_9 with long N-terminal domain (N1 and N2 modules) and GH13_8 with short N-terminal domain (N2 module only, also known as CBM48 domain) (Leggio et al., 2002; Suzuki and Suzuki, 2016). Interestingly, GBE with longer N-terminus tends to transfer shorter oligosaccharide chains during glycogen branching process (Binderup et al., 2002; Jo et al., 2015; Wang et al., 2015). A recent bioinformatics analysis also indicated that there might be a new GH13 GBE with even longer N-terminus than GH13_9 GBE due to the multi-time duplication of the CBM48 domain (Wang et al., 2019a). For a summary of GBE types, please refer to Figure 4. However, there is currently no experimental evidence supporting the existence and role of the new GH13 GBEs. Thus, it would be interesting to investigate into this issue. Moreover, a deep understanding of how the sequence and structure of GBEs influences glycogen primary structure is worthy of further exploration.

In mammals and human beings, glucose metabolism in diabetic liver is dysregulated and in pathological state (Petersen et al., 2017), which leads to abnormal structure of glycogen particles. For example, according to the subtle structure analysis of fluorophore-assisted carbohydrate electrophoresis (FACE), diabetic liver glycogen was consistently observed to have a comparatively longer chain length distribution when compared with glycogen in healthy liver (Li and Hu, 2019; Wang et al., 2020a; Nawaz et al., 2021). In a recent review, Li and Hu argued that the abnormally long chain length distribution in diabetic liver was controlled by the imbalance of enzymatic activities between glycogen synthase (GS) and GBE, which could also serve as a therapeutic target for diabetes (Li and Hu, 2019). Wang et al. (2020a) studied the influences of glucose concentration on glycogen chain length distribution via the model organism *Caenorhabditis elegans*, according to which, high glucose diet did facilitate the shift of chain length distribution toward longer regions (Liu et al., 2020a). However, the molecular mechanism behind the primary structural change is not clear yet. Metabolically speaking, longer chain length distribution might contribute to the fast degradation of glycogen particles due to the reasons stated above, which could then exacerbate diabetic hyperglycemia, leading to uncontrolled release of glucosyl residues. However, more substantive evidence is needed to support this hypothesis. In other clinical situation, abnormal chain length is also associated with many severe diseases, such as Lafora Disease and Adult Polyglucosan Body Diseases (APBD). In these diseases, longer than normal chain lengths reduce the solubility of glycogen molecules, resulting in a pathogenic build-up of insoluble glycogen, hence the formation of polyglucosan bodies (Nawaz et al., 2021).

### Density Distribution of Glycogen β Particles

Except for physical features such as particle size, chain length and branching degree, the density of glycogen β particle is another important feature that plays essential roles in physiological functions. Multiple methods have been developed to measure the density distribution of nanoparticles, including polysaccharides like glycogen. For example, as a common benchtop technique, differential centrifugal sedimentation (DCS) is used for analyzing high resolution size distributions and particle densities (Minelli et al., 2018). Although DCS is useful for comparing qualitative differences between samples if one sample is heterogeneous with lighter and heavier particles, the effect of separation is a combination of the size, density, and shape of particles, which makes it unlikely for a quantitative measurement of size distribution and density (Gilbert and Sullivan, 2014). Size exclusion chromatography (SEC, a type of gel-permeation chromatography, GPC) with differential refractive index (DRI) and multiple-angle laser light scattering (MALLS) detectors is another commonly used method for the analysis of size distributions of the weight-average molecular weight and of the molecular density as functions of the size of the glycogen hydrodynamic radius *R*_h_ (Wang et al., 2019b).

Recently, by using the synchrotron SAXS technique, Besford et al. (2015) shows that glycogen particles follow specific density distributions. The technique, involving the scattering of X-rays from all electrons in a sample, allows identification of the pair-distance distribution (i.e., the distribution of all distances between electrons in a single average particle). In addition, Zhang et al. (2018) uses the Monte Carlo model to simulate glycogen structure. The result shows that the radial density reaches maximum close to the center of glycogen particles, which is consistent with random growth pattern of glycogen particles (Zhang et al., 2018). Even more recent work by Besford et al. (2020) has again shown these same density distributions (pair-distance distributions) for commercial glycogen beta particle preparations that matches the density observed previously.

According to previous studies, chain length distribution could also be one of the determinants for glycogen β particle density. That is, glycogen particles with small averaged chain length are much denser than those with large averaged chain length (Wang et al., 2020b). Moreover, higher density may exert spatial hindrance for glycogen phosphorylase and glycogen debranching enzyme to function, which thus leads to slow release of glucosyl residues, that is, slow degradation (Wang et al., 2020b). It should be noticed that the primary structure of bacterial glycogen β particles is not as ideal as the theoretical models. There are a good portion of long chains with DP greater than 20 glucosyl residues (Wang et al., 2019b). What are the branching degrees of these longer chains? Where do they distribute within the glycogen β particles, the inner tiers or outmost tiers? how do they influence the density of glycogen β particles. These are the questions that are no doubt interesting and need to be solved. For example, through SEC-DRI-MALLS technique, it is shown that the densities of glycogen β particles are heterogeneous in both prokaryotes (*Escherichia coli*) and eukaryotes (*Caenorhabditis elegans*, *Mus musculus*) (Hu et al., 2018; Wang et al., 2019b; Liu et al., 2020a). However, the relationship between chain length distribution and glycogen β particle density conflicts with theoretical prediction. That is, *E. coli* glycogen β particles extracted via the harsh alkaline method had shorter average chain length and lower density than those extracted via the mild centrifugation method (Wang et al., 2019b). A possible explanation is that other molecules such as proteins might be embedded into glycogen β particles with longer average chain length, which increases the density of particles as a whole. However, more experimental evidence is required to validate the hypothesis.

### Glycogen-Associated Proteins

Glycogen metabolism occurs through the concerted action of a defined group of enzymes, among which a portion of proteins are associated with glycogen particles that are defined as glycogen proteome (Parker et al., 2007). Initially, Meyer et al. (1970) identified a glycogen-protein complex during studying glycogen particles purified from rabbit muscle, in which binding proteins such as glycogen phosphorylase, phosphorylase kinase and phosphatase were detected. Since glycogen particles are spatially and temporally regulated, the granules experience a dynamical life in the cell and the associated proteome is extremely difficult to define (Stapleton et al., 2010). In order to solve this interesting question, Stapleton et al. (2010) purified glycogen particles from mouse and rat liver and dissected the binding proteins via mass spectrometry and bioinformatics analysis. A total of 14 proteins were identified that were found in both mouse and rat glycogen proteomes, which included a variety of functional proteins such as glycogenin-1, glycogen synthase (liver form), and starch-binding domain-containing protein 1 (Stapleton et al., 2010). Further exploration of these binding proteins in the glycogen proteome facilitates the understanding of the dynamic nature and cellular distribution of glycogen particles. In addition, glycogen proteomes in adipocyte and brain were also studied, which led to the discovery of novel glycogen-protein associations, indicating cell-specific differences in glycogen metabolism (Stapleton et al., 2013; Brewer and Gentry, 2019). Although the fine structure of glycogen molecules in *C. elegans* is recently reported, there is still no study focusing on its glycogen proteome (Liu et al., 2020a). Considering the important roles that glycogen plays in *C. elegans*, such as longevity and oxidative stress resistance (Seo et al., 2018), it would be a great privilege to have an understanding of the corresponding its glycogen proteome. As for glycogen associated proteins in prokaryotes, there is currently no touch in this topic yet probably because of the presumably simpler structure of glycogen particles in prokaryotes than in eukaryotes, which is worthy of further investigation.

## Glycogen α Particles

### Glycogen α-Particles Across Species

#### Prokaryotes

As a widely accepted opinion, cellular life is divided into three domains, that is, archaea, bacteria, and eukarya (Woese et al., 1990). Experimental studies showed that glycogen particles were identified in each life domain, which suggested that glycogen metabolism evolved before the separation of the three life domains (König et al., 1982). Interestingly, glycogen α particles have also been observed in archaea, bacteria, and eukarya, which suggested that the formation of glycogen α particles is evolutionarily conservative across species, though specific formation mechanisms might be different (Wang et al., 2019b). The distribution of energy metabolism pathways in archaea was systematically investigated by Wang et al. (2019c), according to which, halophilic archaea prefer using polyhydroxyalkanoates (PHAs) as an energy reserve while thermophilic archaea prefer synthesizing and utilizing glycogen particles. From the evolutionary point of view, a possible reason that halophilic archaea do not favor glycogen as storage compound is due to its inability in increasing osmotic pressure. For example, *Haloarchaea* requires high inner osmolarity for survival in extremely halophilic environment (Oren, 2013) while cytoplasmic glycogen consists of large amount of glucose without a significant increase in osmolarity (Meléndez-Hevia et al., 2000). As for glycogen structure, TEM image from the thermophilic archaeon *Sulfolobus acidocaldarius* showed glycogen particles with diameter of around 350 nm (König et al., 1982), which confirmed the presence of glycogen α particles. In bacteria, glycogen α particles have been observed in some species, such as *Selenomonas ruminantium* and *Fibrobacter succino*genes (Wang and Wise, 2019). A recent study conducted by Rashid et al. (2016) also confirmed the existence of glycogen α particles in *Mycobacterium tuberculosis*, *Streptomyces venezuelae*, and *Escherichia coli*. Thus, both Gram-positive and Gram-negative bacteria are capable of forming glycogen α particles. However, the specific functions of glycogen α particles in bacteria have not been solved yet and should be investigated in future studies.

#### Eukaryotes

**FIGURE 5:**
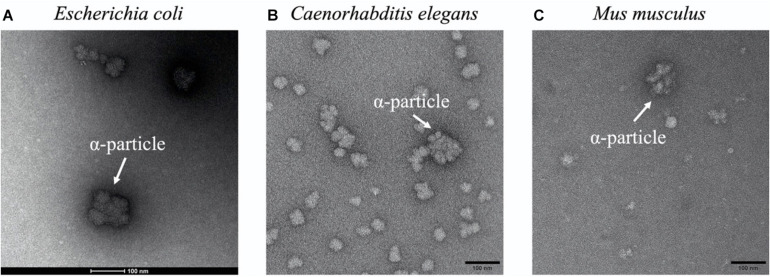
Representative glycogen α particles in TEM images. **(A)** *Escherichia coli*. **(B)** *Caenorhabditis elegans*. **(C)** *Mus musculus*. α particles were designated in white arrows.

The presence of glycogen α particles is also widely identified in eukaryotic species, such as fungi, worms, animals, and human beings. For example, the fungal genus *Sporothrix* includes some pathogenic species such as *Sporothrix schenckii* and *S. brasiliensis*, that could cause sporotrichosis, in which glycogen α particles were identified in the cytoplasm close to the cell wall and the plasma membrane via high-pressure freezing transmission electron microscopy (HPF-TEM) (Reynolds et al., 2018). Since glycogen α particles mainly distributed at the budding pole of these yeast cells, it was hypothesized that they serve as a source of glucose for cell wall enzymes (Reynolds et al., 2018). Similar distribution of glycogen α particles was also observed in another fungus *Candida albicans* (Yamaguchi et al., 1974; Reynolds et al., 2018). However, why α particles are preferred than β particles during glycogen accumulation is not discussed in these studied. In addition, mutated plants such as Sugary-1 mutant maize are able to accumulate a glycogen-like polysaccharide known as phytoglycogen, in which α particles have also been reported (Powell et al., 2015). Due to the convenient availability of phytoglycogen in large quantity, it has been widely used in cosmetic and food industries (Liu et al., 2020b). As for worms, they are widely used in biological science as animal models, among which *C. elegans* is particularly interesting due to its production of a large number of offspring (Meneely et al., 2019). A series of studies have linked glycogen metabolism with oxidative stress resistance and longevity in *C. elegans* (Seo et al., 2018). However, the fine molecular structure of glycogen particles in *C. elegans* was not elucidated until its recent characterization by Liu et al. (2020a). According to the study, glycogen in *C. elegans* has a large proportion of β particles through SEC analysis and α particles are occasionally spotted in the TEM image (Figure 5; Liu et al., 2020a). *C. elegans* may constantly need instant energy for activities such as movement and reproduction. Considering that β particles are a more efficient energy supply due to its faster degradation than α particles, it is reasonable to postulate that accumulation of glycogen β particles is preferred in this model animal and other similar organisms. Moreover, glycogen structure in human beings and mammals like mouse and rat have also been extensively studied due to its importance in many severe diseases (Deng et al., 2015; Caporali et al., 2016). In these organisms, glycogen α particles distribute in an organ- and/or tissue-specific manner due to the differential requirements of physiological functions, which shall be discussed in details below.

### Glycogen α Particles in Organs, Tissues, and Cells

Glycogen was initially discovered in animal liver by Bernard (Young, 1957) and later by Revel (1964). Soon after that, Sanson isolated glycogen from spleen, muscle, and kidney of a horse (Brewer and Gentry, 2019). A variety of other organs and tissues also contain glycogen particles, such as heart (Mowry and Bangle, 1951; Besford et al., 2012), brain (Brewer and Gentry, 2019), retina (Samorajski et al., 1965), pancreas (Brereton et al., 2016), uterus (Dean, 2019), fallopian tubes (Dean, 2019), vagina (Amabebe and Anumba, 2018), embryos (Daimon, 1977) and adipocytes (Ceperuelo-Mallafré et al., 2016). In addition, some types of cells accumulate glycogen for different but important physiological purposes too, which include but not limited to macrophages (Ma et al., 2020), lymphocytes (Astaldi and Verga, 1957), erythrocytes (Moses et al., 1972), neurons (Saez et al., 2014), oncocytes (Zois and Harris, 2016) and astrocytes (Robert et al., 2001; Castejon et al., 2009). In specificity, glycogen have been intensively studied in liver and muscle due to their importance in blood glucose control (Han et al., 2016) and energy supply (Ørtenblad et al., 2013), respectively. In adipose tissue, glycogen is present in low quantities, the accumulation of which is actually harmful and associated with obesity-linked inflammation in humans (Ceperuelo-Mallafré et al., 2016). As for brain glycogen, it was first discovered in normal and diabetic human brain in 1880s (Brewer and Gentry, 2019). However, due to the trace amounts (10-30 mg/100g brain tissue in vertebrates), the study of brain glycogen in terms of its structure, regulation, chemical properties, subcellular distributions, and associated proteins is rather difficult (Brewer and Gentry, 2019). In recent years, glycogen metabolism in brain and neurons is gaining more attentions because of its emerging roles in cognition, learning, and memory (Waitt et al., 2017), which also benefits from the technical development of glycogen quantification, isolation, visualization, and structural characterization. As for kidney and pancreas, glycogen is not normally present or only exist in trace amount; however, in diabetic conditions, considerable amount of glycogen has been observed in both pancreatic β-cells and kidney (Brereton et al., 2016; Wang et al., 2020d). Glycogen also plays important but distinguished roles in different parts of female reproductive system, such as uterus, fallopian tubes, and vagina. In the uterus and fallopian tubes, glycogen is an important source of glucose during early pregnancy (Dean, 2019). In vagina, glycogen is mainly present in vaginal fluids, which is released from vaginal epithelial cells, in order to facilitate the colonization of beneficial *Lactobacillus* species via maintaining low vaginal pH (Fredricks et al., 2014; Amabebe and Anumba, 2018).

As for the presence of glycogen particles in different types of cells, a variety of studies have been conducted. For example, reserved glycogen is reported in macrophages that plays an important function in inflammatory responses through NADPH yield via degradation into glucose-6-phosphate, which could increase the inflammatory survival of macrophages (Ma et al., 2020). As for lymphocytes, glycogen content is normally maintained at very low level (Astaldi and Verga, 1957). However, in some disease conditions such as chronic lymphatic leukemia and lymphosarcoma, high amount of glycogen was reported (Jones et al., 1962), which might support lymphocyte survival and function in hypoxic tissues during inflammation (Lang et al., 2020). As for glycogen structure in lymphocytes, only uniform and low-molecular weight glycogen particles existed, which did not form glycogen α particles (Lutkic and Fister, 1972). It is noteworthy that glycogen was also detected in human and bovine milk; since milk contains white blood cells, glycogen in milk might be present in the liquid and within white blood cells (Naidu and Newbould, 1973; Matsui-Yatsuhashi et al., 2017). Glycogen metabolism was also found in normal erythrocytes, in which glycogen content was maintained at insignificant level; however, in certain conditions where glycogen phosphorylase and/or glycogen debranching enzymes were deficient in the red blood cells, the amount of glycogen could reach several hundred times of the normal level (Moses et al., 1972). In terms of cancer cells, dysregulation of glycogen metabolism is a recently discovered feature in many tumor types such as kidney, ovary, lung, bladder, liver, blood, and breast, which leads to a variety of pathophysiological conditions (Zois and Harris, 2016; Khan et al., 2020). Further studies reveal that glycogen plays an important role in the aggressiveness and survival of cancer cells, which could be used as new targets for cancer therapy (Dauer and Lengyel, 2019).

Although glycogen is widely distributed in different body parts and cell types of an animal or a human being, glycogen α-particles are mainly identified in the liver, the structure and properties of which have recently been extensively studied due to its linkage with hyperglycemia in diabetes (Deng et al., 2015). Glycogen α-particles were also reported to be occasionally seen in some other organs and tissues, such as muscle (rat muscle and insect flight muscle), heart, retina, and brain (Brewer and Gentry, 2019). However, the specific functions and formation mechanisms of these α-particles are not clear. In addition, the structural features of glycogen molecules, together with their associated proteins, have been not characterized in many tissues and cells yet. Considering that the physiological roles of glycogen particles are tightly correlated with glycogen structures, a complete atlas of the distribution of glycogen α- and β-particles, together with the dynamical change of α-particle structure and glycogen proteomes, is needed for understanding glycogen metabolism and functions. Thus, there are still much work to do toward this direction.

### Diurnal Change of Glycogen α-Particle Structure in Mice

Circadian clock is an endogenous timing system for organisms to adapt to daily environmental changes. Similar to many metabolic pathways, glycogen synthesis and degradation follow a daily rhythm in a variety of species in terms of light-dark cycle and food availability, such as cyanobacteria (Shinde et al., 2020), mice (Hu et al., 2018), and human beings (Macauley et al., 2015). It is commonly known that glycogen amount increases after each meal and then decreases to fasting levels thereafter in human beings (Macauley et al., 2015). It is also well known that, in healthy mouse liver, glycogen level reached the maximum during the night period and decreased to the minimum during the light period (Reznick et al., 2013). However, how the structure of glycogen α particles in the liver changes is not clear until recently (Deng et al., 2015; Hu et al., 2018). In a well-designed study, Hu et al. (2018) thoroughly compared the fragility and stability of glycogen α particles in healthy mice C57BL/6JNju and type 2 diabetes mellitus (T2DM) mice C57BL/6JNju-*db*/*db*, according to which, healthy liver glycogen was fragile to DMSO at synthesis stage (8 pm, 0 am, 4am at night time) but stable at degradation stage (8 am, 12 pm, and 4 pm at day time) while diabetic liver glycogen was always fragile. Another study used two type 1 diabetes mellitus (T1DM) models, NOD/ShiLtJNju mice and streptozotocin-injected C57BL/6J mice, exploring the dynamic change of glycogen α-particle stability and fragility, which generates a similar result as that in T2DM. Thus, the impairment of glycogen structure is common to both type 1 and type 2 diabetic mice (Hu et al., 2019). It was then concluded that high blood-glucose level and/or insulin deficiency could be the cause of α-particle fragility (Hu et al., 2019). However, more evidences shall be provided at molecular level in order to validate these claims.

### Dynamic Change of Glycogen α-Particle Structure in *E. coli*

Since glycogen α particles were previously identified in prokaryotes, it would be interesting to investigate whether the structure of α particles also alters between fragility and stability during different stages of glycogen metabolism. It has been reported that most bacteria accumulate glycogen in stationary phase when nitrogen source is limited while carbon source is abundant (Wilson et al., 2010; Wang and Wise, 2011). However, through comparative analysis of glycogen content curves, it was noticed that glycogen metabolism in *Escherichia coli* had two stages, synthesis stage and degradation stage (Strydom et al., 2017; Wang et al., 2020c), which could be used to study the structural alteration of glycogen α particles. By using *E. coli* BL21(DE3) as a model organism, we recently revealed that glycogen α particles in bacteria followed a similar pattern of structural change as in healthy liver, that is, fragile during synthesis stage and stable during degradation stage (Wang M. et al., 2021); in addition, fragile glycogen particles tend to have longer chain length distribution than stable glycogen particles (Wang M. et al., 2021), which is also consistent with previous results in healthy and diabetic mice (Hu et al., 2018). Thus, it would be possible that high glucose concentration triggers glycogen synthesis pathway, which leads to the up-regulation of catabolic enzymes and down-regulation of anabolic enzymes, vice versa. Thus, the balance between catabolic and anabolic enzymes could be a key factor for glycogen stability and fragility in bacteria.

Taken together, the large, rosette-shaped glycogen α particles have been reported in many organisms from prokaryote and eukaryotes with subtle morphological differences. Physiological functions of glycogen α particles are intensively investigated and debated at current stage. Meanwhile, glycogen α particles have also been reported in a variety of organs, tissues, and cell types. Whether any other part of an animal body accumulates α particles requires further investigations with the assistance of optimal glycogen extraction methods and high-resolution analytical instruments (Wang Z. et al., 2021), the answer to which could improve our understandings of glycogen functions in these body parts. Interestingly, it is recently revealed that glycogen α particles have two states: stability and fragility. Since the structure of glycogen α particles experiences constant alterations between stable state and fragile state, the dynamic change of glycogen structure is also scrutinized in both prokaryotes and eukaryotes, which could provide a better understanding of the structural features of glycogen α particles.

## Molecular Mechanisms of Glycogen α-Particle Formation and Fragility

Although the structure of glycogen α particles plays important roles in controlling the releasing rate of glucosyl residues, how glycogen α particles assemble in eukaryotes and prokaryotes are currently not known and are waiting for satisfactory explanations, not even mentioning its stable and fragile features (Deng et al., 2015; Wang et al., 2019b). In eukaryotes, liver glycogen metabolism is a highly-regulated and extremely-sophisticated process, which involves gene expression, hormone regulation, enzyme cooperation, and protein phosphorylation (Petersen et al., 2017). In addition, glycogen-associated proteins form the glycogen proteome (Stapleton et al., 2010), which makes the assembly of glycogen α particles more complicated. In prokaryotes, mechanisms of glycogen α-particle formation might be different from eukaryotes due to the relatively simpler metabolism processes. Thus, the formation of glycogen α particles might be a type of convergent evolution due to general polymer constraints (Wang et al., 2019b). In this section, we went through some proposed formation mechanisms of glycogen α particles, which may facilitate our understanding of the fragile and stable natures of glycogen α particles.

### Binding Mechanisms of Glycogen α Particles

The question of how glycogen β particles aggregate to form glycogen α particles have not been solved yet. Possibilities include van der Waals force, hydrogen binding, hydrophobic interaction, glycosidic linkage, chain length entanglement, disulfide bridge, and other covalent or strong non-covalent bonds involving proteins (Orrell et al., 2008; Sullivan et al., 2012; Powell et al., 2015). Drochmans first proposed that glycogen particles had a two-state structure: α-particle and β-particle, and β particles aggregated to form rosette-shaped α particles (Drochmans, 1962). Soon after that, Orrell et al. (2008) reported a detailed characteristics of undegraded glycogen, and discussed the possible formation mechanisms of glycogen α particles with fellow scientists in a symposium, where Krebs suggested that the association and dissociation of β particles might involve enzyme-mediated formation and cleavage of chemical bonds (Lumsden, 1965). In another study, Lutkic and Fister correlated rat liver glycogen structure with glycogen level, according to which, the average chain length of glycogen particles increased with increased glycogen concentration, and high concentration of liver glycogen led to aggregation of smaller β particles (Lutkic and Fister, 1972). In addition, they proposed two molecular mechanisms to explain the agglomeration of β particles: 1) increased average chain length, and 2) budding on the surface of the present β particles to permit development of new β particles (Lutkic and Fister, 1972), which is also known as the crowding/budding model (Sullivan et al., 2012; Powell et al., 2015). Although it was postulated that glycogen contained proteins for a very long time, there was no solid evidence until the study by Krisman and Barengo (1975). Further studies demonstrated that initiation of glycogen synthesis needed the participation of a protein primer that was later known as glycogenin (Rodriguez and Whelan, 1985; Whelan, 1986).

Further studies confirmed that glycogen particles were coupled with a more versatile set of proteins (Stapleton et al., 2010, 2013). Thus, the existence of protein-glycogen complex provided the possibilities that the aggregation of β particles could be due to the interactions among glycogen associated proteins (Chee and Geddes, 1977). Several studies also suggested that liver glycogen α particles could be formed by β particles possibly via covalent linkage (Zeqiraj et al., 2014). Later, Sullivan et al. (2012) used various reagents to disrupt disulfide bonds, hydrogen bonds, and hydrophobic interactions; however, no significant change in α particle size was observed, which suggested that these interactions were not responsible for β particle aggregation. Powell et al. (2015) studied size change of glycogen α particles in liver glycogen and phytoglycogen via acid hydrolysis, according to which, formation of α particles in liver glycogen might be due to the linkage of β particles via covalent or strong non-covalent bonds involving proteins. Recently, Besford et al. (2020) shows that glycogen particle sizes and shapes change in the presence of protease enzymes that removes proteins from the particles, which reflects that reduction in surface-bound protein leads to the potential cleavage of alpha particles. However, it is currently not clear which protein(s) are involved in glycogen α particle formation. Because glycogenin, the primer protein of glycogen synthesis, was recently found to be abundantly present on the surface of glycogen particles via proteome analysis (Stapleton et al., 2010), it was postulated as the potential “glue” between β particles (Sullivan et al., 2012). However, previous gene-knockout experiment undertook in yeast showed that glycogenin was actually not essential for glycogen accumulation in eukaryotes, according to which glycogen biosynthesis appeared to be random in the glycogenin null mutant (Torija et al., 2005). Thus, the frequency of glycogen synthesis depends on the genetic background of the null glycogenin mutant. Another recent study also found that the rodent model with glycogenin-deficiency can still form glycogen α particles in the muscle tissue (Testoni et al., 2017). However, when glycogenin was absent in the model, glycogen accumulation was enhanced while glycogen structure was considered as abnormal (Testoni et al., 2017). In specificity, the accumulated glycogen is less homogeneous in size and shape than normal muscle glycogen while some glycogen particles have a fibrillar structure (Oldfors, 2017). Thus, the formation mechanism of glycogen α particles still requires further investigations.

### Molecular Mechanisms of Glycogen α-Particle Fragility

Since the formation mechanism of glycogen α particles has not be elucidated yet, it is rather difficult to understand its fragile nature. So far, there is no much work done to solve the issue. Previous studies via electron microscopy and opalescence curves have revealed that glycogen α particles dissociate to β particles at lower pH, which implicates that “protein glue” might be responsible for glycogen α particle fragility (Drochmans, 1962; Orpin et al., 1976). Recently, Tan et al. (2018) compared glycogen α and β particles in rats, mice, and humans via advanced Sequential Window Acquisition of all Theoretical Mass Spectra (SWATH-MS), which revealed that glycogenin was the only detectable candidate for β-particles linking into large α particles. Based on the discovery, it was then concluded that concentrations of glycogenin on the surface of glycogen particles could vary over feeding cycle, which led to the diurnal change of glycogen α-particle stability (high amount of glycogenin) and fragility (low amount of glycogenin) (Tan et al., 2018). This is currently the only experimental evidence addressing the molecular mechanisms of glycogen α-particle fragility. In another study, Wang et al. (2019b) characterized the glycogen fine molecular structure in *E. coli*, finding that α-particle in bacteria also had two states, fragile and stable. However, neither glycogenin or its homologs have ever been detected in bacteria (Ugalde et al., 2003; Wang et al., 2019b). Considering the vast metabolic and structural differences between prokaryotes and eukaryotes, it was deduced that any organism which needs to store and then release glucose will have similar α and β particle structures: a type of convergent evolution (Wang et al., 2019b). Thus, it might be needed to unravel the mechanisms of glycogen α-particle fragility across species in different life of domains.

Both glycogen accumulation and structure show a diurnal alteration (dark and light cycle) in mouse liver (Doi et al., 2010; Hu et al., 2018). Since liver performs many functions in different phases of the circadian clock (Wang et al., 2018), it is reasonable to postulate that glycogen structure alteration and its regulation are also driven by circadian clock. In specificity, liver glycogen is synthesized during the dark time (feeding time) with glycogen α particles being fragile, and then degraded during the light time (fasting time) with glycogen α particles being mainly stable (Sullivan et al., 2014). So far, some studies have provided molecular explanations for the circadian change of glycogen accumulation in the liver (Doi et al., 2010; Wang et al., 2018). However, there are no explanations for the circadian change of glycogen structures. Could it be possible that the expressions of certain genes (the amounts of certain enzymes) are constantly down-regulated or up-regulated during glycogen synthesis-degradation cycle, which then leads to the rhythmic alteration of glycogen structures between α-particle stability and fragility? If that is the case, would it be possible to answer this question via studying the circadian rhythms of transcriptomes and proteomes in both healthy and diabetic liver? It would also be interesting to compare liver glycogen proteomes at different time points, which may provide direct clues to the structural alterations of glycogen α particles at molecular level.

## Potential Drug Target for Diabetic Therapy

The persistent fragility of glycogen α particles in diabetic liver is considered as a pathophysiological marker, which also suggests the existence of potentially novel drug targets for diabetic therapy. Glycogenin is currently suspected to be a candidate drug target since it is postulated that its concentrations may alter glycogen fragility and stability (Tan et al., 2018). Through over- or under-expression studies of the glycogenin gene in animal models, it could then answer the question whether the level glycogenin would be responsible for glycogen structural variations. In addition, it was found that glycogen average chain length was longer in diabetic conditions than that in normal conditions (Hu et al., 2018). After long-term fasting, it was shown that glycogen particles in diabetic liver had more shorter chains, which could be resistant to further degradation (Wang et al., 2020a). Thus, glycogen structural fragility might be associated with longer average chain length. It is well known that glycogen branching enzyme could break α-1,4-glycosidc linkages within glycogen particles and transfer the truncated oligosaccharides to the same or neighboring chains as branches via α-1,6-glycosidc linkages (Wang et al., 2019a). Thus, up-regulation of glycogen branching enzyme level in diabetic liver might increase the stability of glycogen particles. After an intensive literature review, Li and Hu proposed that the decreased activity of glycogen synthase and the increased activity of glycogen branching enzyme could increase shorter chains in glycogen particles and stabilize glycogen α-particle fragility; thus, GS/GBE ratio might serve as a therapeutic target for diabetes (Li and Hu, 2019).

Previous studies also postulated that high blood glucose and/or insulin deficiency may play important roles in glycogen structural fragility (Hu et al., 2019). Thus, another question to ask is whether it is possible to reverse glycogen α particles from fragile state to stable state via lowering blood glucose level? Several studies have attempted to repair glycogen α-particle fragility by using well-known anti-diabetic drugs (Li et al., 2019; Liu et al., 2020c,d). In specificity, Li et al. (2019) first checked the influences of active components from four anti-diabetic traditional Chinese medicines (TCMs) on the repairment of glycogen structural fragility, which includes astragalus polysaccharide (APS), berberine (BBR), panax notoginseng saponins (PNS) and pueraria flavonoid (PF). The study confirmed that three components, APS, BBR and PF, could strengthen glycogen fragility in diabetes while reducing blood glucose level (Li et al., 2019). However, no molecular mechanisms were explored. In another study, Liu et al. (2020d) used *Dendrobium officinale* polysaccharide (DOP) to treat T2DM mice constructed via high-fat diet and streptozotocin injection, which not only reduced hyperglycemia by improving hepatic glucose metabolism but also stabilized liver glycogen fragility. In particular, molecular studies indicated that DOP could lower serum glucagon level and reduce glucagon combination with glucagon receptor, which then inactivated adenylyl cyclase and reduced the expression of cAMP-dependent protein kinase (PKA), leading to glycogen phosphorylase suppression and glycogen synthase activation (Liu et al., 2020d). In a follow-up study, Liu et al. (2020c) further confirmed that both metformin and berberine could stabilize glycogen structure in the T2DM *db/db* mice through reducing the level of glycogen phosphorylase via the cAMP/PKA signaling pathway and decreasing the affinity of glycogen phosphorylase with glycogen particles. Thus, glycogen phosphorylase might also be a good candidate for restoring glycogen structure impairment.

## Conclusion

Glycogen is an important polysaccharide that is widely present in both prokaryotes and eukaryotes. Due to the sophisticated regulation and heterogeneous nature of glycogen particles, there are still many unknowns in terms of glycogen metabolism and structure. Recently, a series of studies discovered that glycogen α particles have two states, that is, fragility and stability, in different species. In addition, delicate studies revealed that fragility of glycogen α particles was associated with the development of diabetes in animal models, which suggested that novel drug targets might exist for diabetic treatment, hence the possibility of developing novel anti-diabetic drugs. However, the molecular mechanisms of glycogen particle assembly and structural fragility are not solved yet. In this review, we thoroughly examined the recent progress in the field of glycogen α particles. The distribution of glycogen α particles from prokaryotes to eukaryotes was reported, which suggested a common evolutionary driving force for α-particle formation. Thus, uncovering the mechanisms of glycogen α-particle assembly and fragility in prokaryotes like *E. coli* and lower life forms such as *C. elegans* could be able to help us understand the similar processes in animals and human beings. Moreover, the presence of glycogen α and β particles in different organs, tissues, and cell types was also discussed, which emphasized the importance of glycogen structures in its functions. In addition, the dynamic nature of glycogen particle, such as chain length distribution, glycogen associated proteins, and structural fragility were also addressed in both animals and bacteria, which provided possible explanations for glycogen α-particle fragility at molecular level, such as glycogenin concentration and GS/GBE ratio. Finally, effects of common anti-diabetic drugs such as metformin and berberine on diabetic liver glycogen structure were reviewed, which indicated that the repairment of glycogen α-particle fragility was achievable. Thus, further studies like network pharmacology could be conducted to identify the common targets of these drugs, which may provide clues for their therapeutic effects on glycogen α-particle fragility. In sum, although some progresses have been made in terms of understandings glycogen fragility, more work is needed to gain a better picture of the molecular assembly of glycogen α particles, together with its physiological functions in diabetes.

## Author Contributions

LW, XZ, and Q-HL: conceptualization and validation. LW and Q-HL: methodology. LW, Q-HL, and J-WT: formal analysis and investigation. LW, XZ, and P-BW: resources. Q-HL, J-WT, and M-MW: data curation. LW, Q-HL, J-WT, and P-BW: writing – original draft preparation. LW, Q-HL, M-MW, and XZ: writing – review and editing. LW and Q-HL: visualization. LW and XZ: supervision. LW: project administration. LW and XZ: funding acquisition. All authors have read and agreed to the published version of the manuscript.

## Conflict of Interest

The authors declare that the research was conducted in the absence of any commercial or financial relationships that could be construed as a potential conflict of interest.
